# Utilizing Multiple Behavioral Endpoints to Identify Negative Control Chemicals in a Larval Zebrafish Behavior Assay

**DOI:** 10.3390/toxics13090727

**Published:** 2025-08-29

**Authors:** Bridget R. Knapp, Deborah L. Hunter, Jeanene K. Olin, Stephanie Padilla, Kimberly A. Jarema

**Affiliations:** 1 Oak Ridge Institute for Science and Education (ORISE) Research Participation Program Hosted by EPA, Center for Computational Toxicology and Exposure, Biomolecular and Computational Toxicology Division, Rapid Assay Development Branch, U.S. Environmental Protection Agency, Research Triangle Park, NC 27711, USA; 2 Center for Computational Toxicology and Exposure, Biomolecular and Computational Toxicology Division, Rapid Assay Development Branch, U.S. Environmental Protection Agency, Research Triangle Park, NC 27711, USA; 3 Center for Public Health and Environmental Assessment, Immediate Office, Program Operations Staff, U.S. Environmental Protection Agency, Research Triangle Park, NC 27711, USA

**Keywords:** behavior, behavioral neurotoxicity, neurodevelopmental toxicity, benchmark concentration, developmental toxicity, negative control chemicals, positive control, zebrafish

## Abstract

Identifying reliable negative control compounds is essential for determining the sensitivity and specificity of screening assays. However, well-characterized negative controls for developmental neurotoxicity behavioral assays in larval zebrafish (*Danio rerio*) are lacking. This study evaluated nine chemicals with no reported evidence of mammalian developmental neurotoxicity, and a positive control (fluoxetine) for developmental and neurodevelopmental (i.e., behavioral) toxicity in zebrafish. Embryos were exposed to each chemical (≤100 µM) during development, 0–5 days post-fertilization (dpf), then assessed as larvae (6 dpf) using a locomotor behavior light–dark transition test. Behavior was analyzed using two methods: (1) the traditional method, comparing the average total distance moved, and (2) a 13-endpoint approach analyzing 13 aspects of the locomotor profile. Results showed that ibuprofen, omeprazole, and fluoxetine induced developmental toxicity (teratogenesis), with fluoxetine also causing behavioral neurotoxicity. Behavioral effects of developmental exposure to selegiline hydrochloride depended on the analysis method. Exposure to the other six chemicals (D-mannitol, glycerol, L-ascorbic acid, metformin hydrochloride, saccharin, and sodium benzoate), as well as ibuprofen or omeprazole, did not produce behavioral effects using either analysis method. Identifying negative control chemicals is essential for evaluating behavioral alterations precipitated by unknown substances and will assist with screening new chemicals for neurodevelopmental toxicity.

## 1. Introduction

Exposure to environmental chemicals during gestation and childhood may impact neurodevelopment [1,2,3,4]. According to the Toxic Substances Control Act (TSCA) chemical substance inventory (https://www.epa.gov/tsca-inventory, last accessed on 15 May 2025), there are roughly 86,000 chemicals in U.S. commerce, many of which lack animal studies for repeat-dose toxicity. Developmental neurotoxicity data are even more scarce, with less than 150 chemicals tested on laboratory mammals following standard guidance for developmental neurotoxicity studies from either the Environmental Protection Agency (EPA) or the Organisation for Economic Co-operation and Development (OECD) [5]. This is especially concerning because the developing nervous system may be more sensitive to chemical exposures than a fully developed adult nervous system, as reviewed in [6]. Given the lack of developmental neurotoxicity data and the potential consequences to human health and the environment, there is an urgent need for higher-throughput assays to assess the developmental neurotoxicity potential of environmental chemicals with an impetus for rapid testing in alternative, vertebrate models, such as zebrafish [7,8,9].

Zebrafish (*Danio rerio*) have been used as an efficient alternative in vivo model for developmental neurotoxicity screening [7,8,10,11,12,13,14,15,16]. Zebrafish are easy to house and maintain, plus their high fecundity and rapid development allow for high-throughput testing. Unlike many cell-based in vitro models, larval zebrafish are metabolically competent (i.e., able to metabolize xenobiotic compounds), as the general structure of their liver is established by 50 h post-fertilization (hpf), making them an appropriate animal model for screening protoxicants [17,18,19]. Their genome is sequenced and annotated [20], and they possess a thyroid axis and a stress axis similar to humans, both of which are integral in brain development [21,22,23,24,25,26].

Larval zebrafish behavioral assays are commonly used to assess the effects of chemical exposure on the developing nervous system, reviewed in [27,28,29,30]. As new assays are created, it is vital to measure assay sensitivity and specificity to ensure accuracy and precision. To understand an assay’s strengths, weaknesses, and potential for misclassifying a chemical as toxic or non-toxic, both positive and negative controls should be tested. In this instance, a negative control is a chemical that is treated in exactly the same manner as the experimental samples but is not expected to produce a change in the larval behavior, whereas a positive control would be expected to produce a behavioral change. Using negative controls helps determine if the assay produces false-positive results (Type 1 errors), helping to validate the assay.

Controls are important for validating an assay and drawing reliable conclusions from the results. Vehicle controls ensure changes are due to the compound being tested and not the vehicle itself. Positive and negative chemical controls produce previously established anticipated effects, which confirm that the assay is operating correctly. Martin and coworkers conducted a comprehensive literature review to identify chemicals with characteristics that were favorable for use as negative control chemicals in developmental neurotoxicity testing [31]. Even though their analysis was aimed at identifying chemicals for in vitro testing, their review of each chemical was detailed and based on in vivo research, making it an appropriate foundation for identifying negative chemicals for behavioral testing in zebrafish larvae. The goal of the present study was to identify negative control chemicals suitable for the light–dark transition assay for larval zebrafish (e.g., [32]). Nine chemicals defined as “favorable” negative control chemicals, i.e., likely to be developmentally and neurotoxicologically inactive based on available evidence, by Martin and coauthors [31] were first assessed for developmental toxicity in larval zebrafish. Second, each chemical was administered at non-teratogenic concentrations during development, and the effects on the light–dark transition behavioral assay were determined after six days of development. In addition, because predominantly negative results were anticipated, a positive control for both developmental toxicity and behavioral neurotoxicity was included to establish assay credibility.

## 2. Materials and Methods

### 2.1. Zebrafish Husbandry, Spawning, and Embryo Collection

An established population of adult, wildtype zebrafish (*Danio rerio*, AB background strain) was maintained at an American Association for Accreditation of Laboratory Animal Care (AAALAC)-approved animal facility at the U.S. Environmental Protection Agency in Research Triangle Park, NC, USA. The zebrafish breeding colony is descended from two wildtype AB background stocks obtained from two commercial suppliers (Aquatic Research Organisms, Hampton, NH, USA, and EkkWill Waterlife Resources, Ruskin, FL, USA) over 15 years ago. This colony has been periodically outbred with other wildtype strains to maintain genetic variability. Zebrafish were kept at 28 °C in recirculating housing systems (Tecniplast USA, West Chester, PA, USA, or Aquaneering Inc., San Diego, CA, USA) with 7–8 zebrafish/L in 3.5 or 6 L tanks on a 14:10 h light–dark cycle (lights on at 0700 h). The system water consisted of Durham, NC city tap water purified by reverse osmosis and buffered with sea salt (Instant Ocean, Spectrum Brands, Blacksburg, VA, USA) and sodium bicarbonate (Church & Dwight Co., Ewing, NJ, USA) to maintain pH (7.4 ± 0.2) and conductivity (1000 µS/cm ± 200 µS/cm), and maintained within specified ranges of ammonia (0 ppm), nitrite (0 ppm), and nitrate (<39 ppm). Adult zebrafish were fed four times daily, twice with decapsulated artemia (E-Z Egg; Brine Shrimp Direct, Ogden, UT, USA) and twice with Gemma Micro 300 formulated diet (Skretting, Westbrook, ME, USA). All research was approved by the Office of Research and Development’s Institutional Animal Care and Use Committee at the U.S. Environmental Protection Agency in Research Triangle Park, NC, USA (Animal Care and Use Protocols #20-08-003 and #24-05-001).

Mixed-sex adult zebrafish (3 to 15 months old) were set-up to spawn two different ways: (1) the afternoon before embryos were needed, approximately 100 adult zebrafish were added to 17.9 L static spawning tanks with mesh bottom inserts [33]; or (2) a week before embryos were needed, approximately 150 adult zebrafish were added to a 16 L on-rack, flow-through, recirculating spawning tank (Z-Park, Tecniplast, West Chester, PA, USA; https://www.tecniplast.it/usermedia/en/2016/brochures/Z-PARK.pdf, accessed on 28 November 2023), and mesh spawning inserts were added the afternoon before embryos were needed. With both spawning methods, embryos were collected in the morning, approximately 45 min after the room lights came on, and were maintained at 28 °C for 1 to 2 h until they were washed, sorted, and plated.

Embryos were washed twice for five minutes each in 0.06% (*v*/*v*) bleach and 10% Hanks’ Balanced Salt Solution (13.7 mM NaCl, 0.54 mM KCl, 25 μM Na_2_HPO_4_, 44 μM KH_2_PO_4_, 130 μM CaCl_2_, 100 μM MgSO_4_, and 420 μM NaHCO_3_; pH = 7.4 ± 0.2; and dissolved oxygen ≥ 80%; all salts obtained from Sigma-Aldrich, St. Louis, MO, USA; hereafter referred to as Hanks’) and rinsed three times with Hanks’ after each bleach wash [34]. Immediately after washing, healthy embryos with defined cells and intact chorions were selected using an Olympus SZH10 stereo microscope and placed in a glass crystallization dish filled with 150 mL of Hanks’ and allowed to mature at 28 °C. Around 4–6 h post fertilization (hpf), healthy embryos were placed (one embryo per well) into a 96-well mesh microtiter plate (Multiscreen™, Millipore Sigma, Burlington, MA, USA) containing 200 μL of Hanks’ (for developmental toxicity) or 96-square-well plates (Cytiva Microplate Devices Uniplate, Washington, DC, USA) with 200 μL of Hanks’ (for behavioral neurotoxicity). If multiple plates were prepared on the same day, each plate was filled by alternating rows among them to negate any bias among plates. For example, the first two rows were completed on the first plate, followed by the first two rows on subsequent plates, before returning to the first plate to complete the third and fourth rows. The process continued until all plates were filled.

### 2.2. Chemicals and Treatment

Chemicals were obtained from Sigma-Aldrich (St. Louis, MO, USA), unless otherwise noted. Table 1 provides chemical information for the 9 candidate negative chemicals (D-manitol, glycerol, ibuprofen, L-ascorbic acid, metformin hydrochloride, omeprazole, saccharin, selegiline hydrochloride, and sodium benzoate) and the positive control chemical fluoxetine, including the chemical name, CAS number (#), Distributed Structure Searchable Toxicity Substance Identifier (DTXSID) used in the EPA’s CompTox Chemicals Dashboard (https://comptox.epa.gov/dashboard/ v2.5.3, last accessed on 15 May 2025), molecular weight (g/mole), octanol–water partition coefficient (LogKow) predicted median and range, and purity (%). Information about the chemical name, CAS #, molecular weight, and purity were obtained from the manufacturer, while the other chemical information came from EPA’s CompTox Chemicals Dashboard. Stock solutions (25 mM) were made by solubilizing each chemical in dimethyl sulfoxide (DMSO; CAS #: 67-68-5, anhydrous, ≥99.9% pure, Sigma-Aldrich) and then stored at −70 °C until use. To make experimental stock plates, those stock solutions were diluted in 96-well conical bottom microtiter plates (Nunc™ 96-Well Polystyrene Conical Bottom MicroWell™, Thermo Fisher Scientific, Waltham, MA, USA). All stock plates were stored at −70 °C until use.

Chemical stock plates for the developmental toxicity experiments contained 8-point, semi-log, serial dilutions for two different candidate negative control chemicals (0.0075–25 mM, *n* = 4 for each concentration), 100% DMSO as a vehicle control (*n* = 30), and 10 mM fluoxetine (CAS #54910-89-3, ≥98.0% pure, Sigma-Aldrich) as a positive control (*n* = 2). Adding 1.6 µL from the stock plate to the 400 µL Hanks’ on the experimental plate resulted in a 250× dilution of the stock plate solution so that the final concentrations on the embryo experimental plate were 0.03–100 µM of the test chemical in 0.4% DMSO. The final concentration of the vehicle control was 0.4% DMSO, and the final concentration of the positive control fluoxetine was 40 µM in 0.4% DMSO. Historical data in our laboratory has demonstrated that 0.4% DMSO will not cause developmental toxicity or behavioral neurotoxicity in zebrafish embryos/larvae [35], and 40 µM fluoxetine will cause obvious developmental effects [11]. The vehicle control (0.4% DMSO) was included on every plate.

Chemical stock plates for the behavioral experiment each contained the five highest concentrations for one test chemical (*n* = 16 for each concentration) that did not cause death or malformations, as ascertained in the developmental toxicity experiments (see above), with DMSO as the vehicle control (*n* = 16). The behavioral stock plates were prepared using a LabCyte Echo 550 Acoustic Dispenser (San Jose, CA, USA), which allowed for a randomized arrangement of the treatments. Echo Cherry Pick software (v.1.6.2) was used to distribute 250× the desired final concentration of the test chemical in 100% DMSO at a volume of 1.76 µL in each well. All stock plates were stored at −70 °C until use. Before dosing, 220 µL of Hanks’ was dispensed into the Echo stock plate, thereby diluting the original chemical and DMSO 125× (0.8% DMSO; twice the final chemical concentration of each chemical). Then 200 μL of that solution from the Echo stock plate was added to each well on the behavioral neurotoxicity plate already containing 200 µL of Hanks’ and one embryo, resulting in a 1× final concentration of the chemical, with a final DMSO concentration of 0.4% in 400 µL in each well (included on every plate). In general, the dosing range for these experiments was 1–100 µM at semi-log intervals. Lower concentrations were used for ibuprofen, omeprazole, or fluoxetine because of their developmental toxicity.

### 2.3. Developmental Toxicity Experimental Design

**Figure 1:**
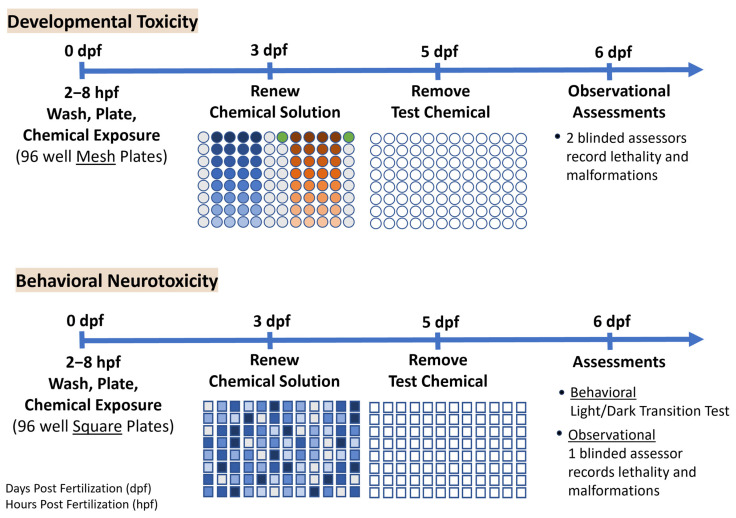
The experimental design presents a timeline for the developmental toxicity and behavioral neurotoxicity experiments from spawning through assessments. Procedures for the two studies were very similar, differing primarily in the type of plate (mesh or square-well), distribution of the chemicals on the plate (by column, or random), chemical renewal process due to plate type, and behavioral assessments included in the behavioral neurotoxicity experiment. Typical developmental toxicity plates included 2 test chemicals, represented by the blue and orange circles (color gradient represents the 8 concentrations for each chemical), light-grey circles represent DMSO vehicle control, and the green circles represent the positive control fluoxetine (40 µM). Only one test chemical was tested on each behavioral neurotoxicity plate, and concentrations were randomly distributed using the ECHO system. The color gradient represents the different chemical concentrations, and light-grey squares represent DMSO vehicle control. The different shades of blue represent an example of how the chemical concentrations might be distributed across the plate by the ECHO, with each shade of blue representing a concentration of the one chemical on that plate.

An overview of the developmental toxicity experimental design is shown in Figure 1. Healthy embryos with intact chorions at approximately 6 hpf were placed in 96-well mesh experimental plates filled with 400 μL of Hanks’, and 1.6 µL of chemical solution was added from the stock plates. Each experimental plate containing the embryos and final concentrations of the chemicals was then covered with a non-adhesive sealer (Microseal ‘A’, BIO-RAD, Hercules, CA, USA) and a lid, wrapped in Parafilm^®^ and placed in an incubator set to 26 °C on a 14:10 h light–dark cycle (Darwin Chambers Co., St. Louis, MO, USA). At 3 days post fertilization (dpf), the chemical solution was renewed by blotting the mesh insert housing the embryos on glass fiber filter paper (Whatman GF/B Paper, Brandel, Gaithersburg, MD, USA), and placing it into a new 96-well receiving plate (Multiscreen^®^ Transport Receiver Plate, Merck Millipore, Burlington, MA, USA) filled with 400 μL of Hanks’. Then, 1.6 µL of solution from the stock plate was added to the embryo experimental plate, which was then sealed as described above, and placed back into the incubator. At 5 dpf, the chemical was removed by blotting the mesh insert with the embryos on filter paper and placing it into a new 96-well receiving plate with 400 µL of Hanks’ without any test chemical or DMSO.

At 6 dpf, the Hanks’ solution was renewed following the same procedure as described above for the day 5 chemical removal process, then two expert observers, blinded to the treatment conditions, independently assessed each larva for lethality, non-hatching, and malformations (i.e., craniofacial, spinal, abdominal, and thoracic malformations along with swim bladder status, abnormal position in the water column, and changes in pigmentation) using a stereo microscope. After the assessments, the larvae were anesthetized using cold shock for 10 min and then euthanized by submersion in ice-cold 20% (*v*/*v*) bleach. If >15% of the control larvae were abnormal on any plate, then that plate was removed, none of the data from that plate were used, and the experiment was repeated. This was a rare occurrence (<5% of the plates).

### 2.4. Behavioral Neurotoxicity Experimental Design

An overview of the behavioral neurotoxicity experimental design is also shown in Figure 1. Healthy embryos with intact chorions at approximately 6 hpf were placed one per well into 96-square-well plates (Cytiva Microplate Devices Uniplate, Washington, DC, USA) with 200 μL of Hanks’ before adding 200 μL from the behavioral stock plates that were prepared using the Echo system. Behavioral plates containing the embryos and final chemical concentrations were covered, sealed, and placed in the incubator following the same process described above for the developmental toxicity experiments. The square-well plates were used to allow more room for locomotor activity and optimize tracking results from the software.

At 3 dpf, the chemical solution in each well was 50% refreshed by first removing 200 μL of the solution in the well and replacing it with 200 μL of the appropriate 1X chemical concentration (0.4% DMSO). At 5 dpf, 97% of the chemical was rinsed out by removing 200 μL of chemical solution and replacing it with 200 μL of Hanks’ solution (no test chemical) five consecutive times. This process of diluting the concentration by 50% on each of the 5 rinses resulted in a final concentration that was approximately 3% of the original concentration. On the morning of 6 dpf, 200 μL of the Hanks’ solution was again refreshed by removing and then replacing with 200 μL of new Hanks’ in each well. Immediately afterwards, the plates were placed in a pre-warmed behavioral testing darkroom, set to 26 °C, and acclimated for at least three hours before behavioral testing began. The assay was conducted with the ViewPoint ZebraBox system (ViewPoint Life Sciences, Montreal, QC, Canada) equipped with LED and infrared lights to record locomotor activity. The behavioral assay began between 1200 and 1300 h so that activity was recorded during the afternoon when baseline activity is most stable [36]. The behavioral protocol was a 20 min acclimation period in the dark (12 lux), followed by a 40 min light phase (3500 lux), and then a 40 min dark phase (12 lux). The light–dark transition test is used extensively throughout the zebrafish research community to assess nervous system function in larval zebrafish [29,37,38,39]. The lower light level was chosen based on the operational boundaries of the Viewpoint system, and the higher light level, as well as the length of the light or dark periods, were chosen to allow the behavior of the control animals during that period to reach and maintain asymptotic levels of locomotor behavior. The luminance was measured using a photometer (Sper Scientific, Scottsdale, AZ, USA). Afterwards, an expert observer blinded to the treatment assessed each larva for lethality and malformations (as described in Section 2.3). Then, the larvae were anesthetized using cold shock for 20 min and, then, euthanized by submersion in ice-cold 20% (*v*/*v*) bleach [40]. As with the developmental toxicity experiments, if >15% of the control larvae were abnormal on the experimental plate, data from that plate were not used, and the experiment was repeated. This was a rare occurrence (<5% of the plates).

Locomotor activity was analyzed from videos using Ethovision XT (Noldus Information Technology, Leesburg, VA, USA) software Version 17. Larvae were detected using a dynamic subtraction method with a minimum object size of 10 pixels. The analysis took place at thirty frames per second, and the total distance moved (cm) was calculated and binned every 2 min. To ensure that only true locomotion was tracked, a minimum distance-moved filter of 0.02 cm was implemented to reduce background noise. Only live, morphologically normal larvae with their swim bladders inflated were included in the behavior analysis.

### 2.5. Data Analysis

Based on historical data in our laboratory, a minimum of 24 embryos per treatment group were used to determine behavioral effects; no outliers were identified or removed. The behavioral data were analyzed using two methods: a traditional analysis of variance (ANOVA) using 2 endpoints and a 13-endpoint analysis.

#### 2.5.1. Statistical Analysis

First, for the traditional method, a repeated measures ANOVA was performed using StatView© (version 5.0.1; SAS Institute, Inc., Cary, NC, USA) to compare the average distance moved in the light and dark periods. The independent variables were chemical concentration and phase (light or dark); the dependent variable was distance moved. If there was an overall effect of chemical concentration or interaction between the light–dark phase and concentration (*p* ≤ 0.05), a Fisher’s least significant difference (LSD) test was used to determine the differences between the control and treatment. Significance level was considered *p* ≤ 0.05 throughout for the parametric analysis.

#### 2.5.2. Concentration–Response Modeling (13-Endpoint Analysis)

Second, a 13-endpoint approach [41] analyzed various aspects of the light and dark phases of the locomotor profile. Equations for each endpoint can be found in Supplementary Table S1. The goal of this approach was to capture more facets and, perhaps, subtler aspects of the behavioral profile of the larval zebrafish. Average speed was calculated by averaging the total activity per 2 min time bin separately in the light (avgSL) and dark (avgSD) periods and combined in both photoperiods (avgST). Habituation was calculated using two methods, each with one endpoint for the light period (hbt1L and hbt2L) and the dark period (hbt1D and hbt2D): habituation 1 was calculated as the slope from the first 2-min time bin to the last 2 min time bin, and habituation 2 was the difference between the slope from the second and last 2 min time bins and the slope from the first and the second-to-last 2 min time bins. In addition, the maximum–minimum ratio (the largest distance moved over the smallest distance moved plus one to avoid dividing by zero) was calculated separately for the light period (RoAL) and for the dark period (RoAD). The startle response was measured using three endpoints: (1) startle acceleration (strtlA), the difference between the first 2 min time bin in the dark and the last 2 min time bin in the light, (2) adjusted startle (strtlAavg), the difference between the first 2 min time bin in the dark and the average speed in the light, and (3) magnitude of startle activity (strtlF), the ratio of the first 2 min time bin in the dark over the last 2 min time bin in the light. Lastly, the dark–light ratio of the area under the curve (AUC_r) was calculated as the dark area under the curve divided by the light area under the curve.

Due to the highly skewed nature of the data, each endpoint was transformed using an optimized Box–Cox power transformation [42] based on the data from a set of recently acquired vehicle controls (641 larvae), which resulted in distributions of each endpoint value that were centered about their mean. Next, tcplfit2, an R package (https://cran.r-project.org/web/packages/tcplfit2/index.html, accessed on 28 November 2023), was used to fit concentration–response curves for each endpoint. The concentration–response relationship was considered active if the hitcall value (a continuous value from 0 to 1) was greater than or equal to 0.9 [43,44]. In tcplfit2, the hitcall value is defined as the product of three proportional weights: (1) goodness of modeling fitting using the Akaike Information Criterion (AIC), (2) at least one median response exceeds a user-defined threshold or cutoff, and (3) the top from the winning model exceeds this threshold. If the curve was considered active based on the hitcall, it was concluded that the chemical caused a concentration-related change in that endpoint, and a benchmark concentration (BMC) was calculated.

## 3. Results

### 3.1. Fluoxetine: Positive Control

**Figure 2:**
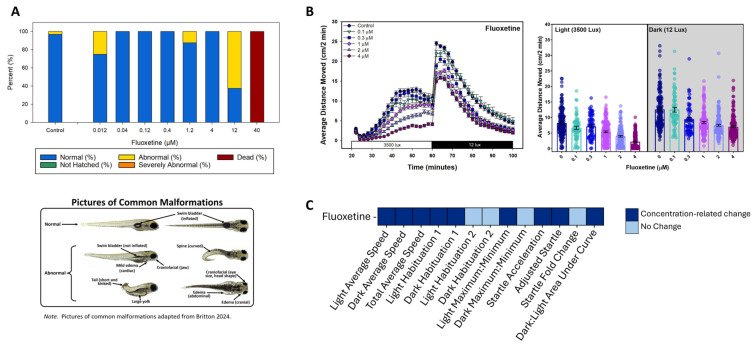
Fluoxetine results following the developmental toxicity and behavioral neurotoxicity experiments. The bar graph in Panel (**A**) presents data from the developmental toxicity observational assessments indicating the status (percent normal, not hatched, abnormal, severely abnormal, and dead); the vertical axis indicates percent (%) affected while the horizontal axis shows fluoxetine concentrations ≤ 40 µM (*n* = 28 for 40 µM and *n* = 8 for all lower concentrations). Beneath the graph are pictures of common malformations previously published by our laboratory [45]. The photos illustrate various malformations and normal-looking images. Panel (**B**) combines the four fluoxetine (0–4 µM) behavioral neurotoxicity experiments together and shows the average distance moved every two minutes in a line graph on the left, and a bar graph on the right with the average distance moved in the light and dark periods separately, with the average for each individual larva represented by the circles. Panel (**C**) presents the behavioral neurotoxicity results from a 13-endpoint analysis in a heatmap, identifying 9 of the 13 endpoints that displayed significant concentration-related changes.

The positive control fluoxetine showed the expected effects in both the developmental toxicity and behavioral neurotoxicity experiments using the traditional ANOVA analysis of the mean activity, as well as the 13-endpoint approach (Figure 2; analysis methods described above). The bar plot in Figure 2A shows the observed status assessments following developmental exposure to ≤40 µM fluoxetine. Exposure to 40 µM resulted in death for all the larvae, while 12 µM caused abnormalities in 62.5% of the larvae, indicating developmental toxicity at both concentrations. Therefore, neither 40 nor 12 µM was used for behavioral neurotoxicity experiments, and only concentrations of 4 µM or less were tested. For developmental toxicity testing, two wells of fluoxetine (40 µM)-treated embryos/larvae were present on each plate as a positive control for developmental toxicity, and the comprehensive assessment results, including chemical and observed status information, are presented in Supplemental Table S2.

With the behavioral neurotoxicity experiments, fluoxetine was tested four times throughout the study. Each exposure resulted in a similar concentration-dependent decrease in activity for both the light and dark phases (Supplemental Figure S1), an overall effect of concentration on activity (Supplemental Table S3), and significant differences between each concentration and the control (Supplemental Table S4). Figure 2B combines all four fluoxetine experiments and shows the average distance moved every two minutes in a line graph, as well as a bar graph of the average distance moved in the light and dark periods separately, with the average for each individual embryo represented by the circles. Exposure to fluoxetine concentrations of 0.3 to 4 µM reduced activity in both the light and dark phases in all four experiments combined (Figure 2B; Supplemental Table S4).

The fluoxetine results from the traditional statistical analysis method (repeated analysis ANOVA) reveal an overall effect of concentration on activity (*p* < 0.0001, Supplemental Table S3). Further analysis using a Fisher’s Least Significant Difference Test showed significant relationships between the total average activity and concentration (*p* < 0.0001, Supplemental Table S4).

**Figure 3:**
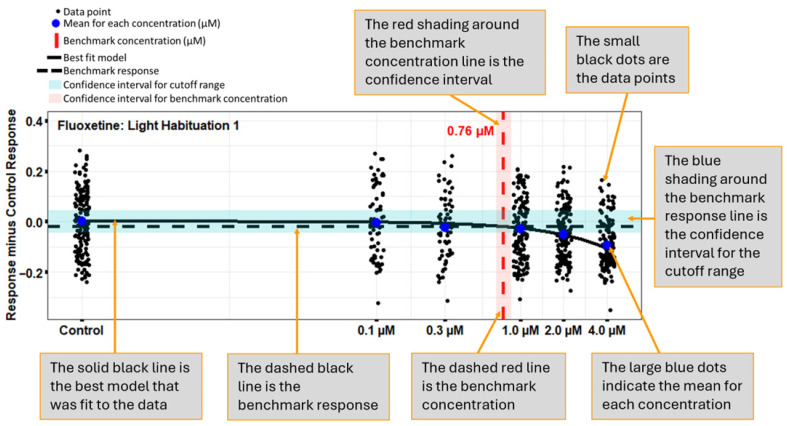
Example of a benchmark concentration (BMC) graph for light habituation 1 following fluoxetine exposure, which depicts the response of each animal (small black dots), the average for each concentration (large blue dots), the benchmark response (dashed black line) with the cutoff range (blue shading), the best-fit model (solid black line), and BMC (µM) (dashed red line) with a confidence interval (red shading).

A heatmap showing results from the 13-endpoint analysis method (Figure 2C) identifies 9 of the 13 endpoints that revealed significant concentration-related changes in the fluoxetine-treated larvae. An example of a benchmark concentration (BMC) graph, using the Light Habituation 1 endpoint following fluoxetine exposure (Figure 3), depicts the response of each animal, the average for each concentration, the benchmark response with the cutoff range, the best-fit model, and the BMC (µM) with a confidence interval. Additional BMC plots for the fluoxetine positive control, and one for selegiline HCL, can be found in Supplemental Figure S2. Several endpoints showed a concentration-related change after fluoxetine exposure, including light average speed (BMC = 0.09 µM), dark average speed (BMC = 0.07 µM), total average speed (BMC = 0.02 µM), light habituation 1 (BMC = 0.76 µM), dark habituation 1 (BMC = 0.01 µM), light maximum:minimum (BMC = 0.21 µM), startle acceleration (BMC = 0.06 µM), adjusted startle (BMC = 0.1 µM), and dark:light area under curve (BMC = 1.5 µM) (Supplemental Figure S2).

### 3.2. Developmental Toxicity Experiments

**Figure 4:**
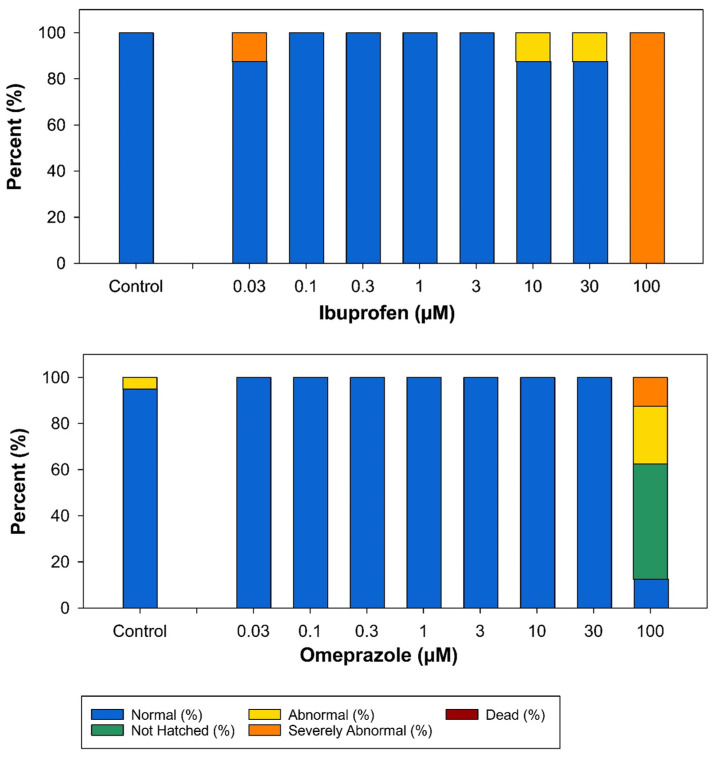
Bar graph showing the developmental toxicity results for ibuprofen and omeprazole at concentrations ≤ 100 µM (*n* = 8 for all concentrations). Observational assessment data is presented for the following percentages: normal, not hatched, abnormal, severely abnormal, and dead. The vertical axis indicates percent affected, while the horizontal axis shows concentration (µM).

Figure 4 presents the observed status assessments for the two candidate negative control chemicals that caused developmental toxicity following exposure to concentrations ≤ 100 µM: ibuprofen and omeprazole. None of the other candidate negative chemicals caused developmental toxicity at concentrations ≤ 100 µM. Exposure to 100 µM ibuprofen caused severe abnormalities in all the larvae (Figure 4 and Supplemental Table S2). Exposure to 100 µM omeprazole prevented 50% of the larvae from hatching, caused abnormalities in 25% of the larvae, and caused severe abnormalities in 12.5% of the larvae (Figure 4 and Supplementary Table S2). Due to this high level of developmental toxicity, 100 µM ibuprofen and omeprazole were not tested in the behavioral neurotoxicity experiments.

### 3.3. Behavioral Neurotoxicity Experiments

**Figure 5:**
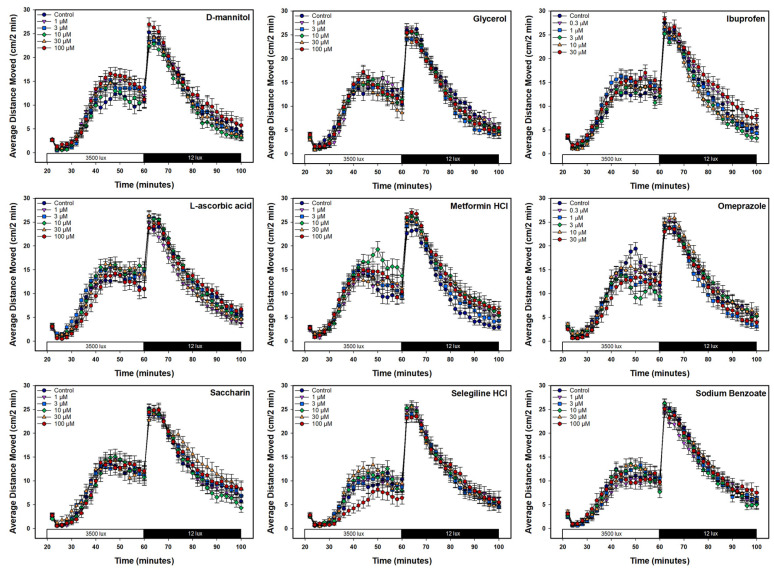
Line graphs for the nine candidate negative control chemicals show the average distance moved every 2 min (vertical axis) during the light–dark assay. Time (min) is shown on the horizontal axis with the first half of the session in the light (3500 lux) and the second half in the dark (12 lux). Vehicle control (DMSO) is represented by the navy-blue line and circle, while each chemical concentration is a different shape and line color.

**Figure 6:**
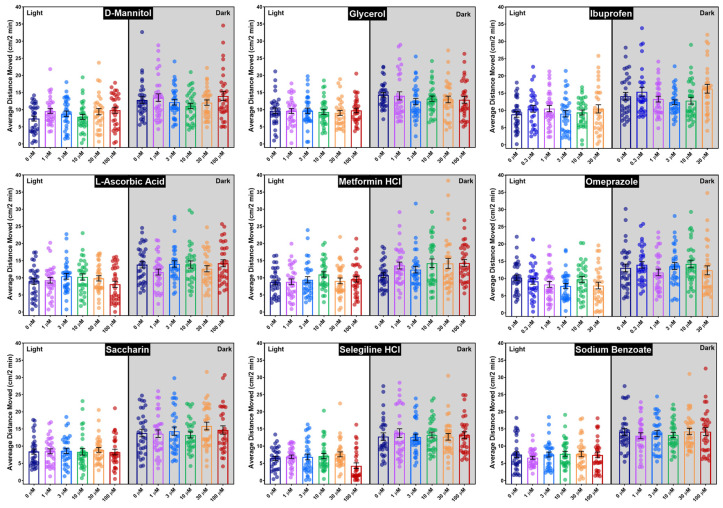
Bar graphs for the nine candidate negative control chemicals show the average distance moved every 2 min (vertical axis) during the light–dark assay. Concentration (≤100 µM) is shown on the horizontal axis, with the light (3500 lux) and dark (12 lux) conditions shown separately, and the average for each individual embryo represented by the colored circles.

**Figure 7:**
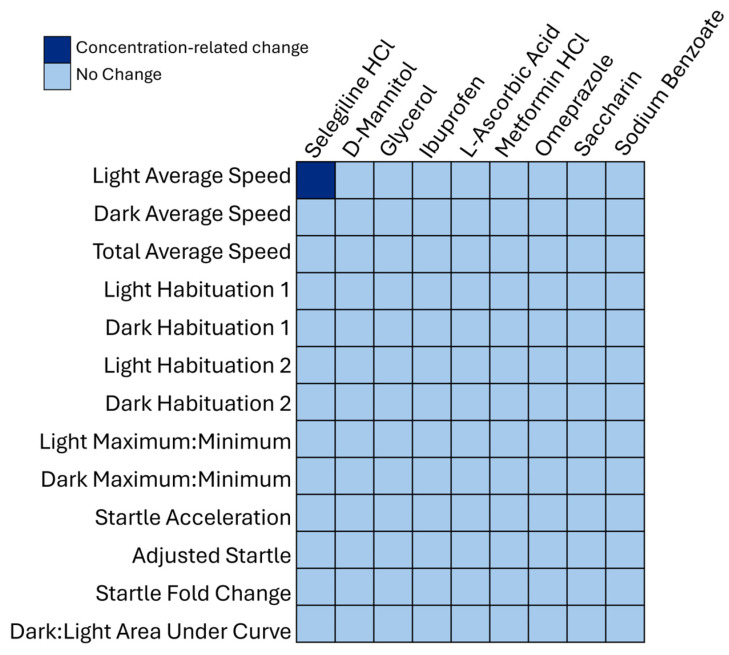
Behavioral neurotoxicity results from a 13-endpoint analysis presented in a heatmap identifying only one endpoint (light average speed for selegiline hydrochloride) that revealed a concentration-related change based on the concentration–response modeling analysis, shaded dark blue. The endpoints that did not show any changes are shaded light blue. The 13 endpoints are listed on the vertical axis and chemicals on the horizontal axis.

Behavioral data for the nine candidate negative control chemicals are shown as a line graph of the average distance moved every 2 min (Figure 5) and a bar plot of the average distance moved in the light and dark periods separately, with the average for each individual embryo represented by the circles (Figure 6). Visual inspection of the data did not reveal any significant differences between the candidate negative control chemicals and the vehicle control (Figure 5 and Figure 6), a finding supported by traditional statistical analyses (ANOVA), which did not result in any statistically significant effects on mean activity following chemical exposure, with *p*-values ranging from 0.10 to 0.84 (Supplementary Table S3). The only chemical that caused a concentration-related change, as revealed with the 13-endpoint analysis method, was selegiline hydrochloride (Figure 7), where a difference in the light average speed (BMC = 89.7 µM, Supplemental Figure S2) was observed. No changes were seen among the other 12 behavioral endpoints with that chemical. Developmental exposure to the other 8 candidate negative control chemicals did not cause changes in any of the 13 behavioral endpoints.

## 4. Discussion

The goal of this study was to identify negative control chemicals appropriate for use in larval zebrafish developmental toxicity and behavioral neurotoxicity chemical screens. Nine chemicals identified by Martin et al. [31] as having no published evidence of activity in mammals for developmental neurotoxicology were tested in zebrafish larvae for developmental toxicity and behavioral neurotoxicity. For assay credibility, fluoxetine served as a positive control. The behavioral data were analyzed using two different methods: (1) a traditional statistical analysis method comparing the test chemical mean activity, with the control mean activity in each respective phase using a repeated measures ANOVA, and (2) a 13-endpoint analysis method calculating a BMC for each of 13 different endpoints gleaned from the profile of the light–dark transition test [41]. Only two of the candidate negative control chemicals, ibuprofen and omeprazole, produced developmental toxicity, and only one of the candidate negative control chemicals, selegiline hydrochloride, produced behavioral neurotoxicity. As expected, the positive control, fluoxetine, consistently caused both developmental toxicity and behavioral neurotoxicity.

Overall, our results were mostly consistent with other published studies, many of which tested much higher concentrations of these chemicals and still failed to find effects. There were, however, some studies where effects were detected at lower concentrations, but factors including differences in assay protocol, duration of chemical exposure, or dechorionation status may explain the differences in results. The specific studies are mentioned below.

Our results show that the candidate negative control chemical ibuprofen produced developmental toxicity, but not behavioral neurotoxicity, which can be compared in more detail to other published reports. In the current study, ibuprofen exposure was developmentally toxic at 100 µM. Many similar studies that tested ibuprofen concentrations ≤ 100 µM found decreased hatching [46,47,48,49,50], which we did not observe at 100 µM. Similar to our study, however, others have noted tail and head malformations, as well as edema after developmental ibuprofen exposure [46,48,49,51]. In the current study, behavioral neurotoxicity was not noted for ibuprofen following developmental exposure to non-teratogenic concentrations (i.e., ≤30 µM). In contrast, several previous publications reported behavioral effects following ibuprofen exposure at concentrations below 30 µM [47,50,52,53]. This could be explained by significant differences in behavioral experimental design or the failure to exclude larvae presenting with malformations (including swim bladder non-inflation).

Our results show that the candidate negative control chemical omeprazole produced developmental toxicity, but not behavioral neurotoxicity, which can also be compared to prior reports. Only two previous studies addressed the developmental toxicity or behavioral neurotoxicity of developmental exposure to omeprazole in zebrafish. Selderslaghs and coworkers [54] reported results similar to the current study, noting that developmental exposure to omeprazole at or above 14.5 µM produced teratogenic effects, but no effects of omeprazole on locomotor behavior were reported at non-teratogenic concentrations. In contrast, another study [55] found omeprazole exposure inhibited pigmentation at concentrations of 30 or 60 µM, whereas the current study did not note abnormal pigmentation in the omeprazole-treated larvae.

For the other seven chemicals, L-ascorbic acid, glycerol, sodium benzoate, saccharin, D-mannitol, metformin hydrochloride, and selegiline hydrochloride, we noted no developmental toxicity at concentrations up to and including 100 µM. In studies testing similar concentration ranges, L-ascorbic acid did not produce developmental toxicity [37,38,56]. Higher concentrations (i.e., millimolar) of L-ascorbic acid, however, produced mortality and inhibited hatching [57]. Most reports indicate that high (i.e., millimolar) concentrations of glycerol were required to produce malformations and/or lethality [58,59,60,61,62]. The sodium benzoate developmental toxicity results were mixed, with one report of micromolar concentrations causing malformations and lethality [63], but others either showed no developmental toxicity in that concentration range [11] or reported that only millimolar concentrations of sodium benzoate caused lethality [59]. Most of the previous studies agree that saccharin or D-mannitol are not developmentally toxic to zebrafish at sub-millimolar concentrations [11,54,64,65,66,67,68,69,70]. In contrast to the metformin hydrochloride results presented in the current paper, some studies have reported that metformin hydrochloride was developmentally toxic to zebrafish embryos/larvae at concentrations in the low- to sub-micromolar range [71,72,73]. Other investigators, however, have either noted a lack of developmental effects in that range [74] or reported that millimolar concentrations of metformin hydrochloride were required to produce developmental toxicity [75,76,77]. In summary, our results, showing no developmental toxicity of seven of the nine candidate negative control chemicals in larval zebrafish at concentrations ≤ 100 µM, generally agree with the published literature.

Selegiline hydrochloride is the only candidate negative control chemical that showed any evidence of behavioral neurotoxicity in our experiments. Developmental toxicity effects were not observed in the current study. However, a behavioral neurotoxicity effect was detected using the 13-endpoint analysis method (i.e., with a change in average speed in the light) but not using the traditional statistical analysis method. Similar studies including this chemical also noted the absence of developmental toxicity in the presence of behavioral neurotoxicity [78,79].

The published literature on larval locomotor behavioral changes produced by developmental exposure to the other eight chemicals (L-ascorbic acid, glycerol, sodium benzoate, saccharin, D-mannitol, metformin hydrochloride, ibuprofen, or omeprazole) showed diverse results. Studies that tested similar concentrations as the present paper observed changes in locomotor activity following L-ascorbic acid exposure [37,38], while Luis and coworkers only noted changes in locomotor activity at L-ascorbic acid concentrations that also elicited significant lethality [57]. Our own previous work [11], along with two other studies [37,54] assessing behavioral neurotoxicity in larvae treated developmentally with saccharin, reported no behavioral changes, whereas another publication reported locomotor changes [38]. Only one study performed a light–dark transition assay in metformin-treated zebrafish and found no changes in locomotor behavior after exposure to millimolar concentrations [76].

Although none of the candidate negative control chemicals appeared to cause any behavioral neurotoxicity when assessed using the traditional statistical analysis method, the 13-endpoint analysis method revealed that selegiline hydrochloride exposure decreased the average speed in the light at the highest concentration (100 µM). In addition, the positive control, fluoxetine, was also confirmed by both methods to be developmentally neurotoxic. The two different analytical methods were not statistically identical, and the method chosen for analysis will likely depend on whether the laboratory is more comfortable with a Type I or Type II error, i.e., the likelihood of detecting false positives or false negatives. The traditional analysis method is more statistically conservative in that the repeated ANOVA accounts for the fact that the same animals were assessed in the light and dark periods and uses a *post hoc* test to correct for multiple comparisons when determining which concentrations are different from the control. The 13-endpoint analysis is more statistically liberal in that it does not correct for multiple comparisons, but it has the advantage of analyzing many different aspects of the light–dark transition test behavioral profile. In general, the traditional analysis as used in our laboratory is likely more prone to false negatives (Type II Errors), whereas the 13-endpoint analysis may be more prone to false positives (Type I Errors). Which analysis is used by a given laboratory depends on its experimental goals. Of course, both analyses can be easily employed and compared, as in the present paper. Unlike the traditional method, another advantage of the 13-endpoint method is that a BMC is computed using the entire concentration–response relationship. This metric can estimate a concentration that is likely to be developmentally neurotoxic even if that specific concentration was not tested experimentally.

This study successfully met the original goal of identifying negative control chemicals for use in a zebrafish light–dark transition assay, as exposure to most of the chemicals tested did not result in developmental toxicity or behavioral neurotoxicity effects and would be appropriate negative controls for this assay.

## 5. Conclusions

Standardizing a set of negative control chemicals is essential to properly evaluate the behavioral alterations precipitated by unknown chemicals and will assist with screening new chemicals for both developmental and neurodevelopmental toxicity. Based on the results of this study, our recommendations would include (1) L-ascorbic acid, glycerol, sodium benzoate, saccharin, D-mannitol, and metformin hydrochloride as appropriate negative control chemicals for both developmental toxicity and behavioral neurotoxicity in larval zebrafish at concentrations of ≤100 µM; (2) omeprazole and ibuprofen could be used as negative control chemicals for behavioral neurotoxicity, but for developmental toxicity care should be taken with the concentrations that are employed; and (3) selegiline hydrochloride could be used as a negative control for developmental toxicity but may not be suitable as a negative control for behavioral neurotoxicity unless used at lower concentrations.

## Figures and Tables

**Table 1:** List of chemicals tested. All were solubilized in DMSO. Each chemical is listed by row. The columns (from left to right) contain the following information: chemical name, CAS number (#), Distributed Structure Searchable Toxicity Substance Identifier (DTXSID), molecular weight (g/mole), octanol water coefficient (LogKow) predicted median and range, and purity (%). Columns with a (*) contain values from https://comptox.epa.gov/dashboard/, v2.5.3, last accessed on 15 May 2025.

Chemical	CAS #	DTXSID *	Molecular Weight (g/mole)	Octanol-Water Partition (LogKow) Predicted Median *	Octanol-Water Partition (LogK_ow_) Predicted Range *	Purity (%)
D-Mannitol	69-65-8	DTXSID1023235	182.2	−3.15	−4.67 to −2.38	≥99.9
Fluoxetine	54910-89-3	DTXSID7023067	309.3	4.16	4.05 to 4.65	≥98.0
Glycerol	56-81-5	DTXSID9020663	92.1	−1.79	−2.32 to −1.65	≥99.0
Ibuprofen	15687-27-1	DTXSID5020732	206.3	3.73	3.37 to 3.79	99.8
L-Ascorbic acid	50-81-7	DTXSID5020106	176.1	−2.15	−3.85 to −1.85	99.2
Metformin hydrochloride	1115-70-4	DTXSID9037246	165.6	−1.12	−2.31 to 0.32	99.9
Omeprazole	73590-58-6	DTXSID6021080	345.4	2.20	2.09 to 3.40	99.9
Saccharin	81-07-2	DTXSID5021251	183.2	0.81	0.45 to 0.91	99.6
Selegiline hydrochloride	14611-52-0	DTXSID9044584	223.8	2.88	0.19 to 2.95	99.8
Sodium benzoate	532-32-1	DTXSID1020140	144.1	0.16	−2.27 to 1.90	99.9

## Data Availability

All raw data are included in the Supplementary Information and will also be uploaded to https://edg.epa.gov/metadata/catalog/main/home.page (accessed on 12 August 2025).

## Supplementary Materials

The following supporting information can be downloaded at https://www.mdpi.com/article/10.3390/toxics13090727/s1: Table S1: Formulas used in the 13-endpoint calculations; Table S2: Developmental toxicity assessment results; Table S3: ANOVA *p*-values; Table S4: Fishers Least Significant Difference *p*-values; Table S5: Raw Data; Figure S1: Data from the four fluoxetine exposures; Figure S2: Benchmark concentrations (BMC).

## Abbreviations

The following abbreviations are used in this manuscript:

AAALAC	American Association for Accreditation of Laboratory Animal Care
ANOVA	Analysis of Variance
AUC_r	Dark–Light Area Under the Curve
avgS_D_	Average Speed in the Dark
avgS_L_	Average Speed in the Light
avgS_T_	Total Average Speed
AZ	Arizona
BMC	Benchmark Concentration
C	Centigrade
CA	California
CAS#	Chemical Abstracts Service Number
cm	Centimeter
D.C.	District of Columbia
dpf	Days Post Fertilization
DMSO	Dimethyl Sulfoxide
DTXSID	Distributed Structure Searchable Toxicity Substance Identifier
EPA	Environmental Protection Agency
FL	Florida
hbt1_L_	Light Habituation 1
hbt1_D_	Dark Habituation 1
hbt2_L_	Light Habituation 2
hbt2_D_	Dark Habituation 2
hpf	Hours Post Fertilization
L	Liter
LogKow	Octanol–Water Partition Coefficient
LSD	Fishers Least Significant Difference
lux	Luminous Flux per Unit Area
µM	Micromolar
µL	Microliter
µS	Micro Siemens
mM	Millimolar
mL	Milliliters
MA	Massachusetts
ME	Maine
MD	Maryland
MO	Missouri
*n*	Number of Independent Observations
NC	North Carolina
NH	New Hampshire
OECD	Organisation for Economic Co-operation and Development
PA	Pennsylvania
ppm	Parts per Million
RoA_D_	Dark Maximum–Minimum
RoA_L_	Light Maximum–Minimum
RTP	Research Triangle Park
strtlA	Startle Acceleration
strtlAavg	Adjusted Startle
strtlF	Startle Fold Change
TSCA	Toxic Substances Control Act
USA	United States of America
UT	Utah
VA	Virginia
