# Supplementary material for: Utilizing Multiple Behavioral Endpoints to Identify Negative Control Chemicals in a Larval Zebrafish Behavior Assay
Source: Toxics. 2025 Aug 29;13(9):727. doi: 10.3390/toxics13090727 (PMC12474258; doi:10.3390/toxics13090727)
Supplement: Supplementary file 1 [file toxics-13-00727-s001.zip › Supplemental Legends.docx]

**Supplemental Legends:**

Supplemental Table 1. Formulas used in the 13-endpoint calculations: light average speed (avgSL); dark average speed (avgSD); total average speed (avgST); light habituation 1 (hbt1L); dark habituation 1 (hbt1D); light habituation 2 (hbt2L); dark habituation 2 (hbt2D); light maximum:minimum (RoAL); dark maximum:minimum (RoAD); startle acceleration (strtlA); adjusted startle (strtlAavg); startle fold change (strtlF); dark:light area under curve (AUC_r).

Supplemental Table 2. The complete developmental toxicity assessment results for all chemicals. Each row contains the location of the larva on the 96-well plate (horizontal row and vertical column), the plate identification number, CAS #, chemical name and concentration (µM) of the chemical and the status of the larva (normal, not hatched, abnormal, severely abnormal, or dead).

Supplemental Table 3. Repeated measures analysis of variance (ANOVA) p-values calculated from data collected during the behavioral neurotoxicity experiments. Only the positive control chemical fluoxetine resulted in a significant overall effect of chemical on activity (≤ 0.05) shown in red.

Supplemental Table 4. Probability values (p-values) from Fishers Least Significant Difference (LSD) test on the average total distance moved data calculated from the four fluoxetine exposures during the behavioral neurotoxicity experiments. Significant values (≤ 0.05) shown in red.

Supplemental Table 5. All behavioral neurotoxicity data presented as one chemical on each worksheet. The average distance moved (cm) for each 2 minute interval, with dark and light periods identified. All concentrations are in micromolar (µM).

Supplemental Figure 1. Data from the four fluoxetine exposures during the behavioral neurotoxicity experiments. Line graphs show the average distance moved every 2 minutes (vertical axis) during the light/dark assay. Time (min) is shown on the horizontal axes with the first half of the session in the light (3500 lux) and the second half in the dark (12 lux). Bar graphs show the average distance moved every 2 minutes (vertical axis) during the light/dark assay. Fluoxetine concentration (≤ 4 µM) is shown on the horizontal axes for the light (3500 lux) and dark (12 lux) conditions separately, with the average for each individual embryo represented by the colored circles.

Supplemental Figure 2. Benchmark concentrations (BMC) for each chemical and endpoint that resulted in a significant concentration-related change as determined by the R package tcplfit2 which was used to fit concentration-response curves for each endpoint from the behavioral neurotoxicity experiments using the 13-endpoint analysis method. A more detailed description of visual elements in the figure can be found in Figure 3.
