# Supplementary figures and images for: Utilizing Multiple Behavioral Endpoints to Identify Negative Control Chemicals in a Larval Zebrafish Behavior Assay
Source: Toxics. 2025 Aug 29;13(9):727. doi: 10.3390/toxics13090727 (PMC12474258; doi:10.3390/toxics13090727)

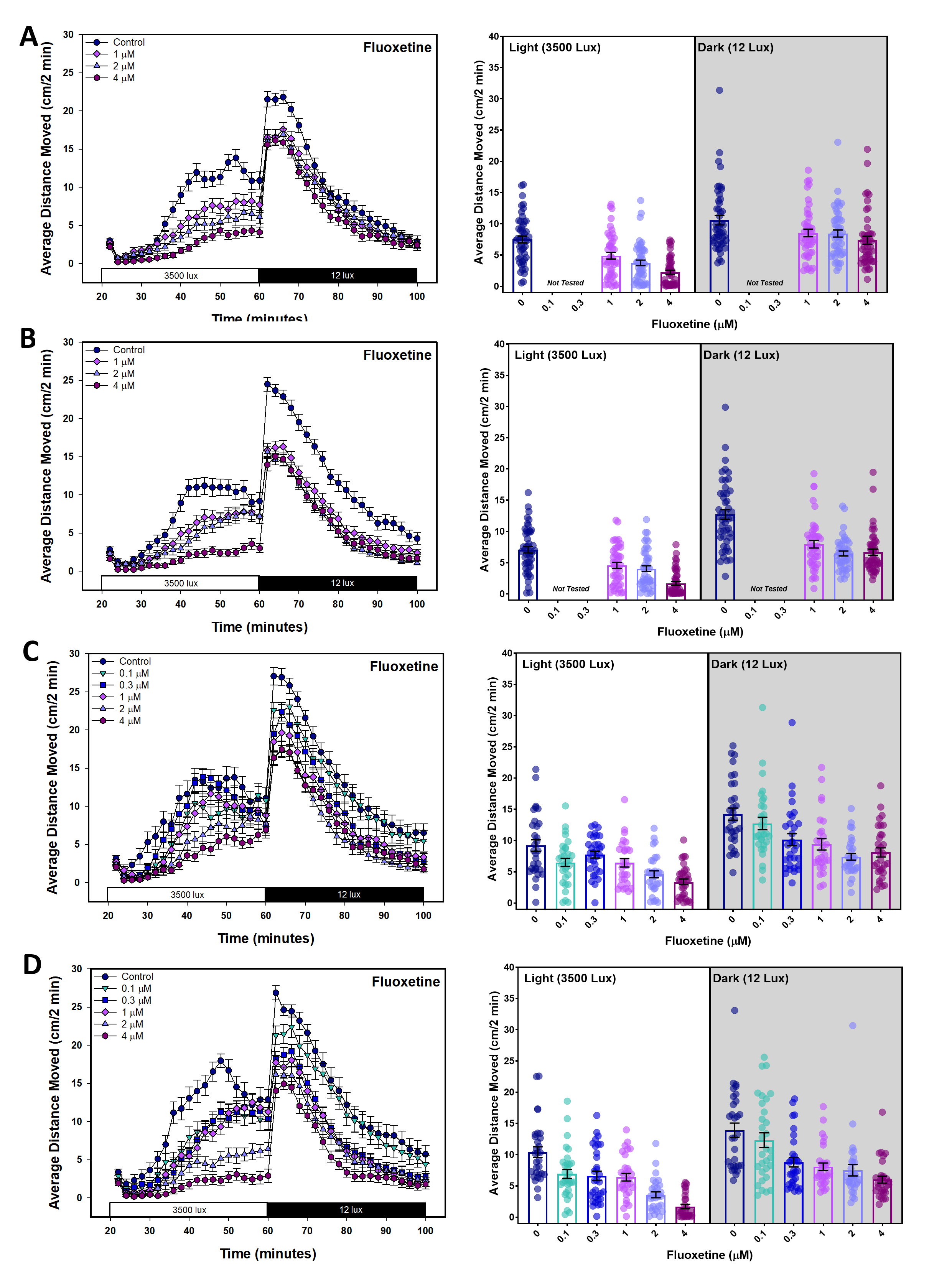

Supplement: Supplementary file 1 [file toxics-13-00727-s001.zip › Suppl Figure 1 Fluoxetine Graphs.png]

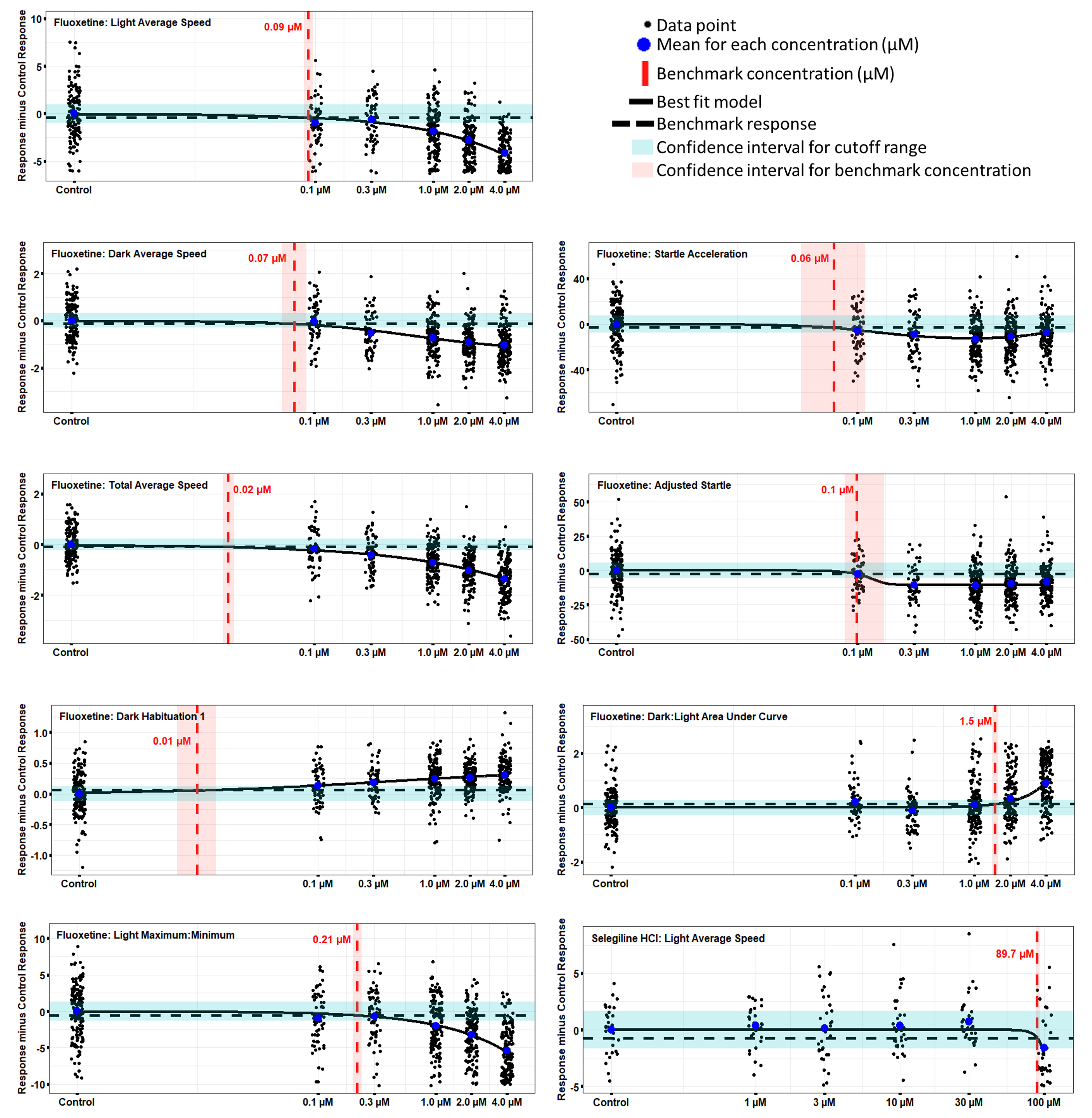

Supplement: Supplementary file 1 [file toxics-13-00727-s001.zip › Suppl Figure 2 BMC plots.png]

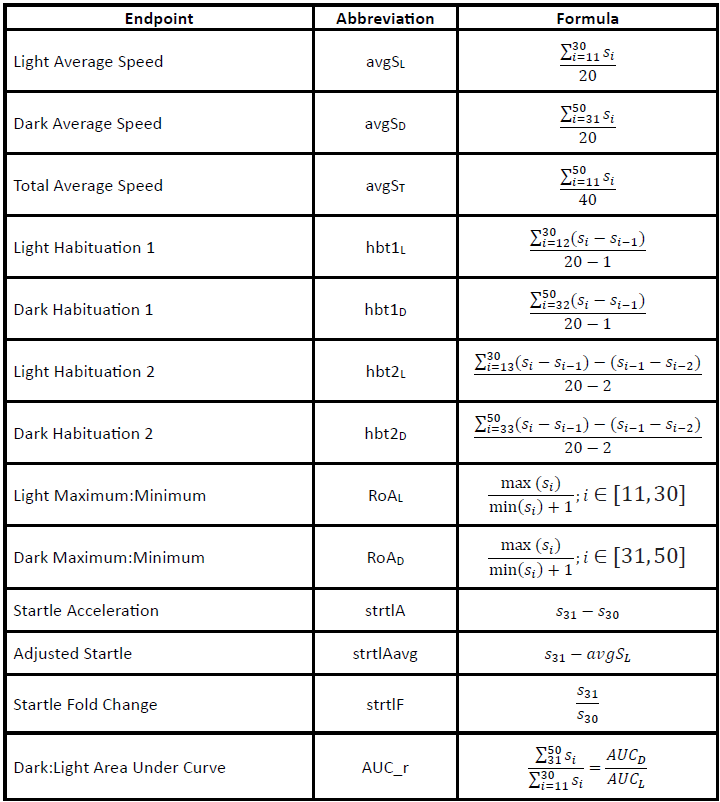

Supplement: Supplementary file 1 [file toxics-13-00727-s001.zip › Suppl Table 1 Formulas.png]

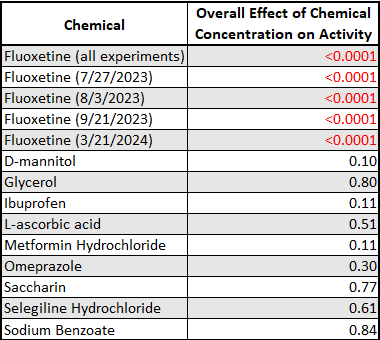

Supplement: Supplementary file 1 [file toxics-13-00727-s001.zip › Suppl Table 3 ANOVA results.png]

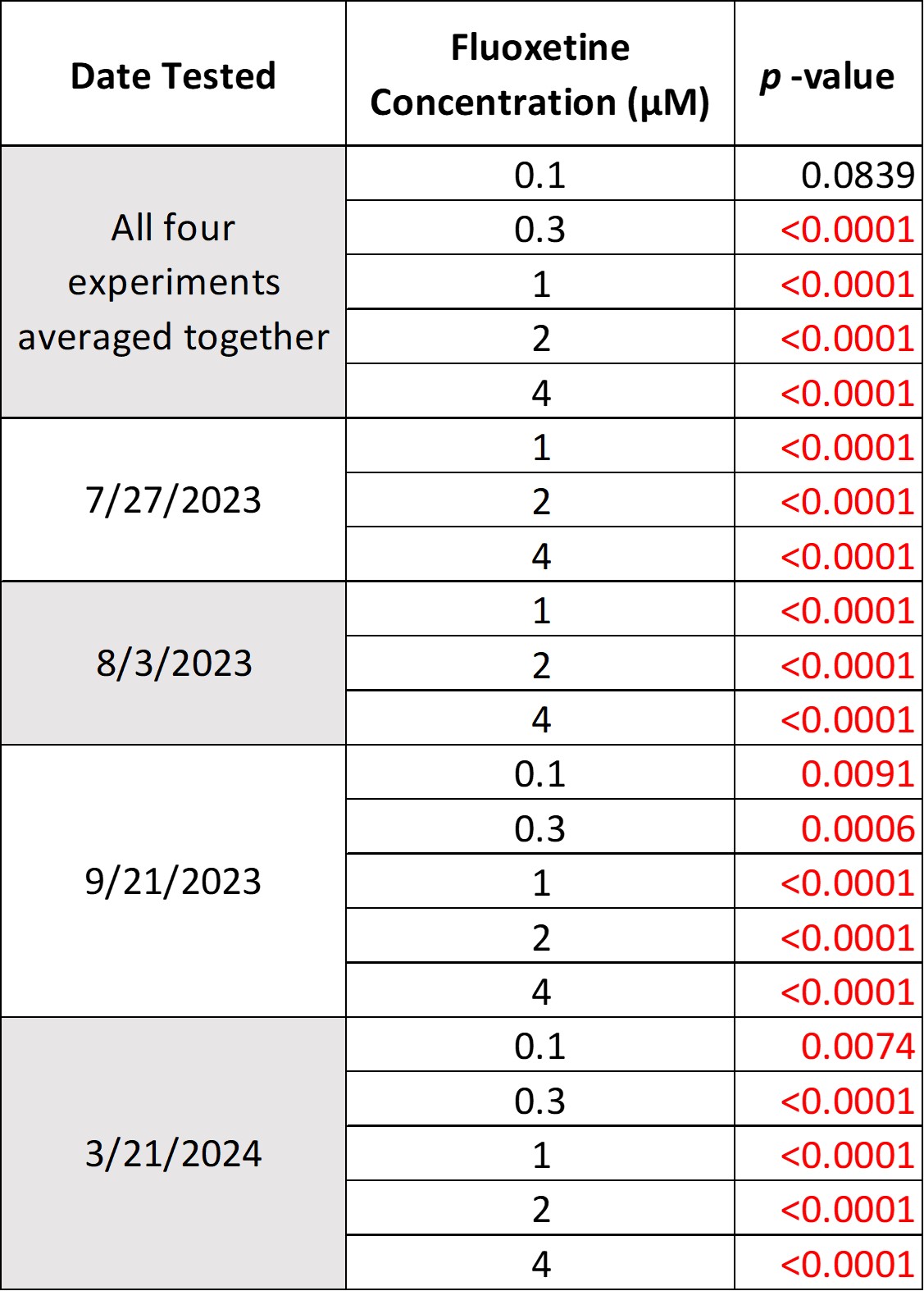

Supplement: Supplementary file 1 [file toxics-13-00727-s001.zip › Suppl Table 4 Post Hoc Analysis.jpg]
